# NLSdb—major update for database of nuclear localization signals and nuclear export signals

**DOI:** 10.1093/nar/gkx1021

**Published:** 2017-11-02

**Authors:** Michael Bernhofer, Tatyana Goldberg, Silvana Wolf, Mohamed Ahmed, Julian Zaugg, Mikael Boden, Burkhard Rost

**Affiliations:** Department of Informatics, I12—Chair of Bioinformatics and Computational Biology, Technical University of Munich (TUM), Boltzmannstrasse 3, 85748 Garching/Munich, Germany; School of Chemistry and Molecular Biosciences, The University of Queensland, Brisbane 4072, Australia; Institute of Advanced Study (TUM-IAS), Lichtenbergstrasse 2a, 85748 Garching/Munich, Germany; Institute for Food and Plant Sciences WZW—Weihenstephan, Alte Akademie 8, 85354 Freising, Germany; Department of Biochemistry and Molecular Biophysics, Columbia University, New York, NY 10032, USA

## Abstract

NLSdb is a database collecting nuclear export signals (NES) and nuclear localization signals (NLS) along with experimentally annotated nuclear and non-nuclear proteins. NES and NLS are short sequence motifs related to protein transport out of and into the nucleus. The updated NLSdb now contains 2253 NLS and introduces 398 NES. The potential sets of novel NES and NLS have been generated by a simple ‘*in silico* mutagenesis’ protocol. We started with motifs annotated by experiments. In step 1, we increased specificity such that no known non-nuclear protein matched the refined motif. In step 2, we increased the sensitivity trying to match several different families with a motif. We then iterated over steps 1 and 2. The final set of 2253 NLS motifs matched 35% of 8421 experimentally verified nuclear proteins (up from 21% for the previous version) and none of 18 278 non-nuclear proteins. We updated the web interface providing multiple options to search protein sequences for NES and NLS motifs, and to evaluate your own signal sequences. NLSdb can be accessed via Rostlab services at: https://rostlab.org/services/nlsdb/

## INTRODUCTION

Eukaryotic cells are characterized by the envelopment of DNA into a membranous compartment, the nucleus (name: Greek *ευ* (eu) = well and *καρυoν* (karyon) = core). Shuttle proteins known as *karyopherins*, namely importins and exportins, facilitate the transport of proteins into and out of the nucleus through nuclear pore complexes (1–4). To identify their cargo, these proteins use specific sequence motifs: so-called nuclear localization signals (NLS) for the import into the nucleus, and nuclear export signals (NES) for the transport out of the nucleus.

NLS motifs vary substantially in terms of length and features (5–7). However, almost all share a simple feature, namely short stretches of mostly basic amino acids with the consensus sequence K-K/R-X-K/R. When a single such NLS on the sequence leads to import into the nucleus, these motifs are referred to as *monopartite*, while *bipartite* NLS often have two monopartite signals separated by a variable linker of typically 9–12 amino acids (8,9). The linker regions can be longer and tripartite motifs exist, as well. Many other types of NLS exist, e.g. the Proline-Tyrosine NLS (PY-NLS), named after the PY group in its motif: R/K/H-X_(2–5)_-P-Y (10).

The classical NES motif contains three to four hydrophobic amino acids, often leucine, and was first identified in HIV-1 (11,12). Several solutions to describing the consensus sequence of NES have been proposed (13–15), but they did not suffice to identify new NES-containing proteins (16).

Public databases such as UniProtKB (17), NESbase (14) and ValidNESs (18) have been able to collect just a few hundred experimentally verified NES and NLS motifs. Machine-learning methods targeted filling the gap, e.g. SeqNLS (19), NucPred (20), NESsential (16) and NLStradamus (21). However, as for every good prediction method, they make mistakes: those designed to predict nuclear proteins might mislabel non-nuclear proteins (false positives), and NES or NLS prediction methods might misplace the signal or miss it altogether (false negatives).

Our group has been developing generic machine learning-based methods predicting localization for almost two decades (22–26). In contrast, we developed NLSdb (27) as a comprehensive and specific database collecting as many experimentally verified NLS in a single resource as possible. In contrast to related resources before and after this, we also considered it to be important to enrich the resource by simple *in silico* analyses. For instance, although good experiments use controls for annotations, they cannot access as comprehensive datasets for controls as we can *in silico*: many motifs published by experimentalists mapped to many non-nuclear proteins, i.e. they were not specific enough (28), and many others mapped only to very few related proteins, i.e. were too specific (28). Experts tried to manoeuvre optimally between these two extremes beginning from 114 NLS carefully collected from the literature. Those were complemented by 194 potential NLS generated by ‘*in silico* mutagenesis’, i.e. mutated and tested by a computational algorithm essentially through iterating over the following simple steps (27,28): if too specific: make more general by shrinking motif, if too unspecific: lengthen motif. All operations (shorten/lengthen) were guided by multiple sequence alignments. Fifteen years later, we have now completed a major update to NLSdb using a similar algorithm, although this time we gave more control to the computer. We have also added NES motifs to the database.

## MATERIALS AND METHODS

### Datasets: nuclear and non-nuclear proteins

We downloaded all proteins from the UniProtKB/Swiss-Prot database (release May 2017), which had manually curated annotations for their subcellular localization (UniProtKB evidence code ECO:0000269). To reduce unrealistic protein fragments, we removed all proteins shorter than 50 residues. We masked low-complexity regions with SEG (29) and removed all proteins that did not have at least one segment of 30 consecutive unmasked residues.

We split the dataset into two distinct sets of nuclear and non-nuclear proteins. Proteins with experimental annotations for both nuclear and non-nuclear subcellular localizations were considered nuclear proteins. Although we previously ignored less reliable annotations (e.g. by similarity), we made an exception: if a protein was annotated experimentally as non-nuclear and it was annotated as nuclear by one of those less-reliable annotations, we removed it from our set. Analyzing the remaining set, we still found pairs of nuclear/non-nuclear proteins that had over 80% pairwise sequence identity. Though, the presence or absence of a single NLS could easily explain why two proteins N and C have >80% PIDE; and because N has the NLS and C does not, only N is nuclear. However, such observations might also suggest mistakes in the annotations. For security, we simply removed all such proteins from the set of non-nuclear proteins (toward this end we applied CD-Hit (30)). Our final datasets contained 8421 nuclear and 18 278 non-nuclear proteins.

Finally, we used UniqueProt (31) (HVAL > 0) to divide the set of nuclear proteins into 801 clusters of similar sequences, hereafter referred to as protein families. Those 801 families were used in the last step of our *in silico* mutagenesis algorithm (below).

### Lists of NLS and NES

Next, we extracted all proteins from UniProtKB/Swiss-Prot (release May 2017) with nuclear export and localization signals (NES and NLS). We kept only proteins with the following evidence codes for their signal annotations: (i) ECO:0000269 (manually curated information for which there is published experimental evidence); (ii) ECO:0000305 (manually curated information which has been inferred by a curator based on his/her scientific knowledge or on the scientific content of an article); (iii) ECO:0000250 (manually curated information which has been propagated from a related experimentally characterized protein); and (iv) ECO:0000255 (manual assertions for information which has been generated by the UniProtKB automatic annotation system or by various sequence analysis programs). This resulted in 529 unique NES and 2362 unique NLS. We enriched this initial set tapping into other online sources, namely through 262 NES from ValidNESs, 80 NES from NESbase and 122 NLS from SeqNLS. Our final datasets contained 788 unique NES and 2466 unique NLS.

### Protocol for *in silico* mutagenesis

Starting with our sets of collected NES and NLS, we applied an *in silico* mutagenesis algorithm to generate new potential signals. We applied the same steps to both sets (NLS and NES, for simplicity referred as ‘signals’ in the following).

(i) To ensure a high specificity for nuclear proteins, we removed all signals from the set that matched any of the non-nuclear proteins or did not match any of the nuclear proteins. To reduce the amount of non-functional residues (i.e. linker regions not related to the NLS), we removed signals longer than 30 residues. Next, we generated all possible variations of the remaining signals that differed by a single amino acid from the original sequence. Thus, for each signal we had 20 times its length variants, including the original one.

(ii) We iteratively applied the following two steps until no new variants could be generated: (a) remove all signals matching non-nuclear proteins or not matching nuclear proteins; (b) generate all possible variants by deleting a single residue, i.e. shortening the signal by one.

(iii) We removed signals that deviated too substantially from the features known for generic signals. We kept only NLS that contained at least three positively charged residues (H, K, R), two if they also included a PY-NLS motif (R/K/H-X_(2–5)_-P-Y). At least one region (two for NLS of 20 or more residues) had to have an overall positive charge (i.e. not cancelled out by negative residues right next to the positive ones). NES were removed if they had fewer than three hydrophobic residues (A, F, I, L, M, V), or if <30% of the signal was hydrophobic.

(iv) To filter out potential signals that are too specific, we kept only those that matched proteins from at least two families (as defined above). We removed redundant signals, i.e. for any pair of signals *S_1_* and *S_2_*, if *S_1_* is a substring of *S_2_*, we kept only the shorter signal *S_1_*.

Note that our final signals by design reached 100% specificity on the set of proteins that we used for development (signals were removed if they match non-nuclear proteins). In order to keep specificity high, we deliberately avoided to combine motifs in regular expressions. For instance, experimentally observing the motifs RKHEL and LEHKR does not imply that all motifs matched by [RL][KE]H[EK][LR] constitute a valid NLS. In particular, LEHEL is unlikely to function as NLS.

### Dataset of whole proteomes

We analyzed the ‘entire proteomes’ for the nine eukaryotic organisms that contributed most to our set of experimentally annotated nuclear proteins (91%, Table 1). These were: *Arabidopsis thaliana, Caenorhabditis elegans, Drosophila melanogaster, Homo sapiens, Mus musculus, Oryza sativa, Rattus norvegicus, Schizosaccharomyces pombe* and *Saccharomyces cerevisiae*. We downloaded the latest reference proteomes (release April 2017) from the European Bioinformatics Institute (EMBL-EBI) (32).

**Table 1.** Proteins with NES and NLS in nuclear protein dataset

*Organism*	*Number of nuclear proteins in NLSdb*	*Proteins with NLS*	*Proteins with NES*
*Homo sapiens* (human)	2163	820 (37.9%)	185 (8.6%)
*Schizosaccharomyces pombe* (fission yeast)	1263	282 (22.3%)	52 (4.1%)
*Arabidopsis thaliana* (thale cress)	1241	430 (34.6%)	37 (3.0%)
*Mus musculus* (mouse)	1011	420 (41.5%)	89 (8.8%)
*Saccharomyces cerevisiae* (brewer's yeast)	1010	294 (29.1%)	33 (3.3%)
*Drosophila melanogaster* (fruit fly)	334	149 (44.6%)	20 (6.0%)
*Caenorhabditis elegans* (roundworm)	273	113 (41.4%)	19 (7.0%)
*Rattus norvegicus* (rat)	237	85 (35.9%)	22 (9.3%)
*Oryza sativa* (rice)	140	46 (32.9%)	5 (3.6%)
Sum nine organisms	7672	2639 (34.4%)	462 (6.0%)

***Organism***: latin (common) names for the nine organisms that contributed the most nuclear proteins (sorted by number of nuclear proteins) to NLSdb (together 7672 proteins in these nine organisms accounted for 91% of all currently known 8421 nuclear proteins); ***Number of nuclear proteins in NLSdb***: gives the number of proteins annotated experimentally as nuclear and retained in NLSdb after applying a variety of filters (*Methods*); ***Proteins with NLS/NES***: numbers and fractions (brackets) of the nuclear proteins that contain at least one NLS or NES from NLSdb.

In addition, we downloaded the subcellular localization annotations for 12 003 human proteins from the Human Protein Atlas (release October 2017) (33,34). However, we only used annotations classified as either ‘validated’ or ‘supported’, and ignored the less reliable ‘approved’ and ‘uncertain’.

## RESULTS AND DISCUSSION

### Database growth

Our *in silico* mutagenesis protocol generated 1177 potential NES and 5189 potential NLS from the initial collection of 788 original NES and 2466 original NLS. Of those, 192 potential NES and 1651 potential NLS matched at least two different protein families. In addition to those, NLSdb includes all original NES and NLS that match nuclear proteins, while not matching non-nuclear proteins. The final list is: 398 NES and 2253 NLS.

In comparison: the original NLSdb contained only 308 experimental and potential NLS, and no NES. Those 308 NLS match 1810 (21%) of the 8421 nuclear proteins, but also 1017 of the non-nuclear proteins. Considering only NLS motifs that exclusively match nuclear proteins, the previous version covered only 174 (2%) proteins. In contrast, the NLS of the new NLSdb cover 2928 (35%) of the nuclear proteins. In other words, the error-free coverage of the new version increased by a factor of 17 (factor of 1.6 if ignoring the errors). This also highlighted why we moved from regular expressions (old version) to lists of signal sequences (new version): regular expressions are prone to quickly becoming too permissive leading to matches in non-nuclear proteins.

### Specificity and sensitivity of NES and NLS

Only 206 of the collected 788 NES (26%) matched at least one of the 8421 nuclear proteins without matching any of the 18 278 non-nuclear proteins. In fact, only 231 NES (29%) matched any experimentally annotated nuclear protein. Similarly, only 639 (26%) of the annotated 2466 NLS matched known nuclear proteins without matching non-nuclear proteins, and only 969 NLS (39%) matched any known nuclear protein. In fact, for most proteins with NES and NLS annotations an experimental annotation as nuclear proteins was missing. Only 17% (754 of 4429) of the proteins we used to extract NES and NLS from UniProtKB/Swiss-Prot were experimentally annotated as nuclear.

According to our protocol (*Methods: In silico mutagenesis*), we retained only the 206 NES and 639 NLS that exclusively matched known nuclear proteins. These motifs matched 191 (NES) and 713 (NLS) of the annotated 8421 nuclear proteins. This suggests that most motifs are too specific, i.e. too long to match any other sequence. While such specific motifs might be biologically meaningful, they are not very helpful to discover motifs in proteins outside of the well annotated families.

The *in silico*-refined signals in NLSdb were both more sensitive (i.e. matching more proteins) and more specific (i.e. matching only nuclear proteins): the 192 NES matched 439 nuclear proteins from 265 families, and the 1651 NLS in 2773 nuclear proteins from 618 families. Thereby the average number of nuclear proteins matched by one motif increased from 1.2 for the original NES to 2.6 for the potential NES and from 1.3 for the original NLS to 3.2 for the potential NLS.

The bias in the dominance of particular organisms to the final set appeared to mirror the bias of experimental biology with most annotations from human, relatively few from plants, and relatively many from yeast (Table 1). However, this bias largely disappeared when considering how many proteins of an organism the NLS/NES matched. For instance, although almost ten times more nuclear proteins from human were annotated than from rat, the NLS from NLSdb matched 38% of the experimentally annotated human and 36% of the experimentally annotated rat proteins, i.e. rather similar fractions of the annotated nuclear proteins. The same held for NES.

**Figure 1.**
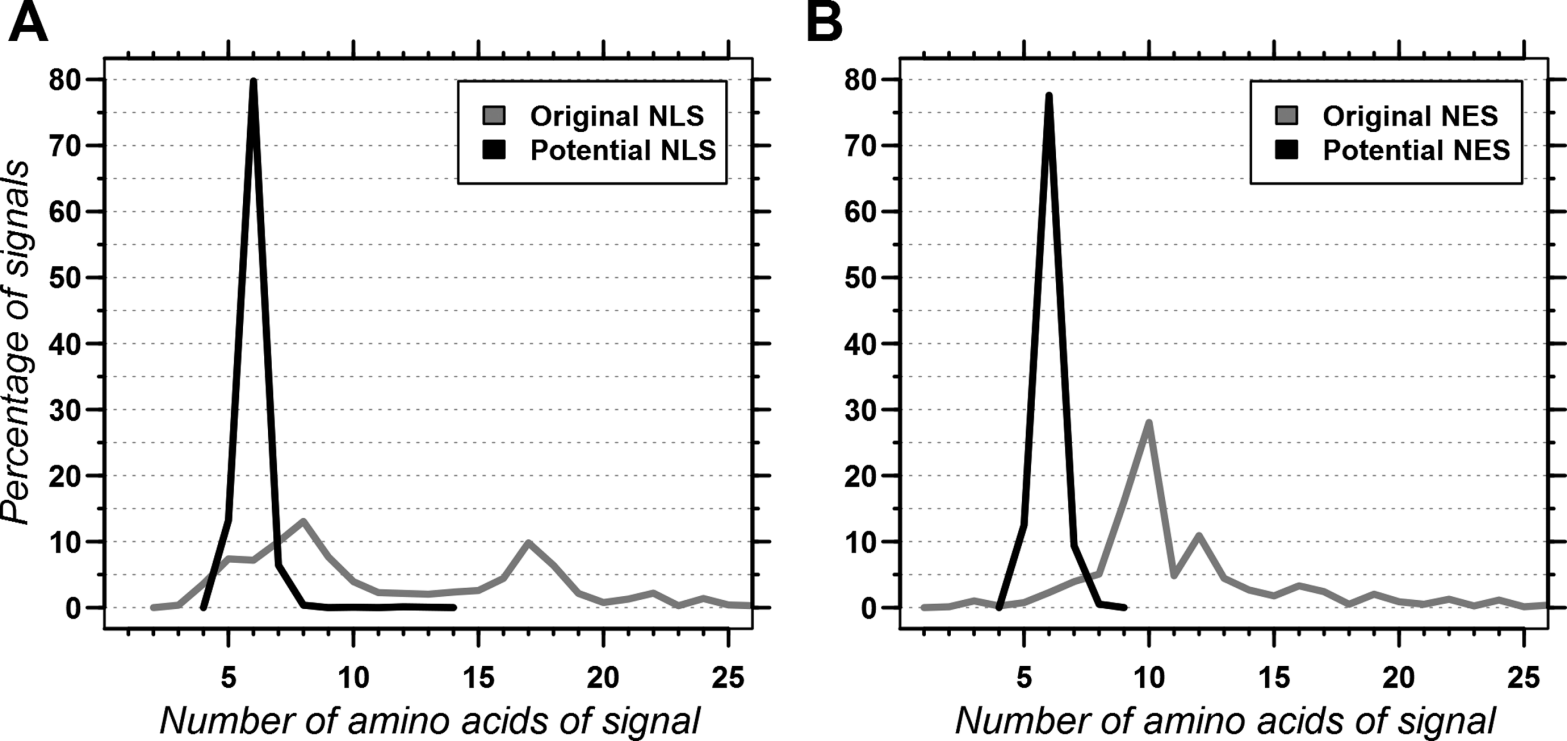
Length distribution of NLS and NES sequences. The graphs compare the length distribution for the original NLS (**A:** gray line; total 2466 NLS) and the NLSdb set of NLS refined through *in silico* mutagenesis (A: dark line; total 1651 NLS), as well as the corresponding distributions for the original NES (**B:** gray line; total 788) and the NLSdb refined set of NES (B: dark line; total 192 NES). Note that motifs with over 25 amino acids were observed, but are not shown in the graphs due to sparseness (total: 156 original NLS, 42 original NES).

The potential NES and NLS in NLSdb were rather short (Figure 1) because our *in silico* mutagenesis protocol can only shorten NES and NLS. Furthermore, we kept only the shorter of two redundant motifs. Almost all of the resulting motifs (NLS and NES) were about six amino acids long. This implies that a match in a protein sequence indicates the site of a NES or NLS, but that the signal tagged by NLSdb might not suffice to facilitate nuclear transport on its own. Instead, the biologically relevant NLS/NES might require additional neighbouring residues to bind to shuttle proteins (karyopherins). For example, two consecutive matches in a protein might indicate a bipartite NLS, while the same signal might match a monopartite NLS in another protein.

### NES and NLS in NLSdb map to about half of all nuclear proteins in popular organisms

The enriched dataset of motifs available through NLSdb matched many proteins in all nine organisms analyzed (Table 2). On average, NLS motifs matched 9–13% of all proteins in each proteome and NES motifs 1–2% (Table 2). Comparing those numbers to the estimated fraction of nuclear proteins predicted by LocTree3 (22), suggested a coverage very similar to that estimated by the set of experimentally verified nuclear proteins (Table 1 versus Table 2). For example, the NLS cover 38% of the experimentally annotated human nuclear proteins, and about 42% of the human nuclear proteins predicted by LocTree3. Overall, the fractions of predicted nuclear proteins suggested that about 30–40% of all nuclear proteins in those nine proteomes were matched by one of the NLS in NLSdb.

**Table 2.** Nuclear signals and proteins in entire proteomes

*Organism*	*Number of proteins*	*Proteins with NLS*	*Proteins with NES*	*LocTree3 (nucleus)*
*Homo sapiens* (human)	21 042	2673 (12.7%)	375 (1.8%)	30%
*Schizosaccharomyces pombe* (fission yeast)	5142	501 (9.7%)	78 (1.5%)	34%
*Arabidopsis thaliana* (thale cress)	27 502	2768 (10.1%)	246 (0.9%)	31%
*Mus musculus* (mouse)	22 262	2684 (12.1%)	358 (1.6%)	30%
*Saccharomyces cerevisiae* (brewer's yeast)	6722	681 (10.1%)	63 (0.9%)	31%
*Drosophila melanogaster* (fruit fly)	13 757	1573 (11.4%)	168 (1.2%)	31%
*Caenorhabditis elegans* (roundworm)	20 057	1759 (8.8%)	170 (0.8%)	27%
*Rattus norvegicus* (rat)	21 412	2555 (11.9%)	359 (1.7%)	28%
*Oryza sativa* (rice)	44 321	4285 (9.7%)	280 (0.6%)	28%

***Organism***: latin (common) names for the nine organisms that contributed the most nuclear proteins to NLSdb (sorted by number of nuclear proteins); ***Number of proteins***: gives the number of proteins found in the ‘entire proteome’ as we accessed it (*Methods: Dataset of whole proteomes*); ***Proteins with NLS/NES***: numbers and fractions (brackets) of the nuclear proteins that contain at least one NLS or NES from NLSdb; ***LocTree3 (nucleus):*** lists the fractions of proteins predicted by our generic machine learning-based method LocTree3 (22) as nuclear.

We tried to validate the specificity of our NLS and NES on the set of potential novel human nuclear proteins by comparing these proteins to annotations from the Human Protein Atlas. Of the 2673 human proteins containing NLS from NLSdb (Table 2), 1853 proteins were not in our dataset of experimentally annotated nuclear proteins from UniProtKB/Swiss-Prot, therefore representing potential novel nuclear proteins. For 516 of them we had subcellular localization annotations from the Human Protein Atlas. There, 407 (79%) of the 516 proteins were annotated as nuclear proteins, implying an error rate of 21% (i.e. proteins containing NLS being non-nuclear). Repeating the same process for the 375 human proteins containing NES (Table 2), we found 65% (34 of 52) of potential novel proteins to be annotated as nuclear. This unfortunately showed that despite our careful approach the signals were not always 100% specific. However, we would like to note that for all 2597 proteins, which were in both the Human Protein Atlas and our entire datasets of experimentally annotated nuclear and non-nuclear proteins, the agreement on being nuclear or non-nuclear was only 90%. Thus, the 79 and 65% should be compared to an upper limit of 90% and not to 100%.

**Figure 2.**
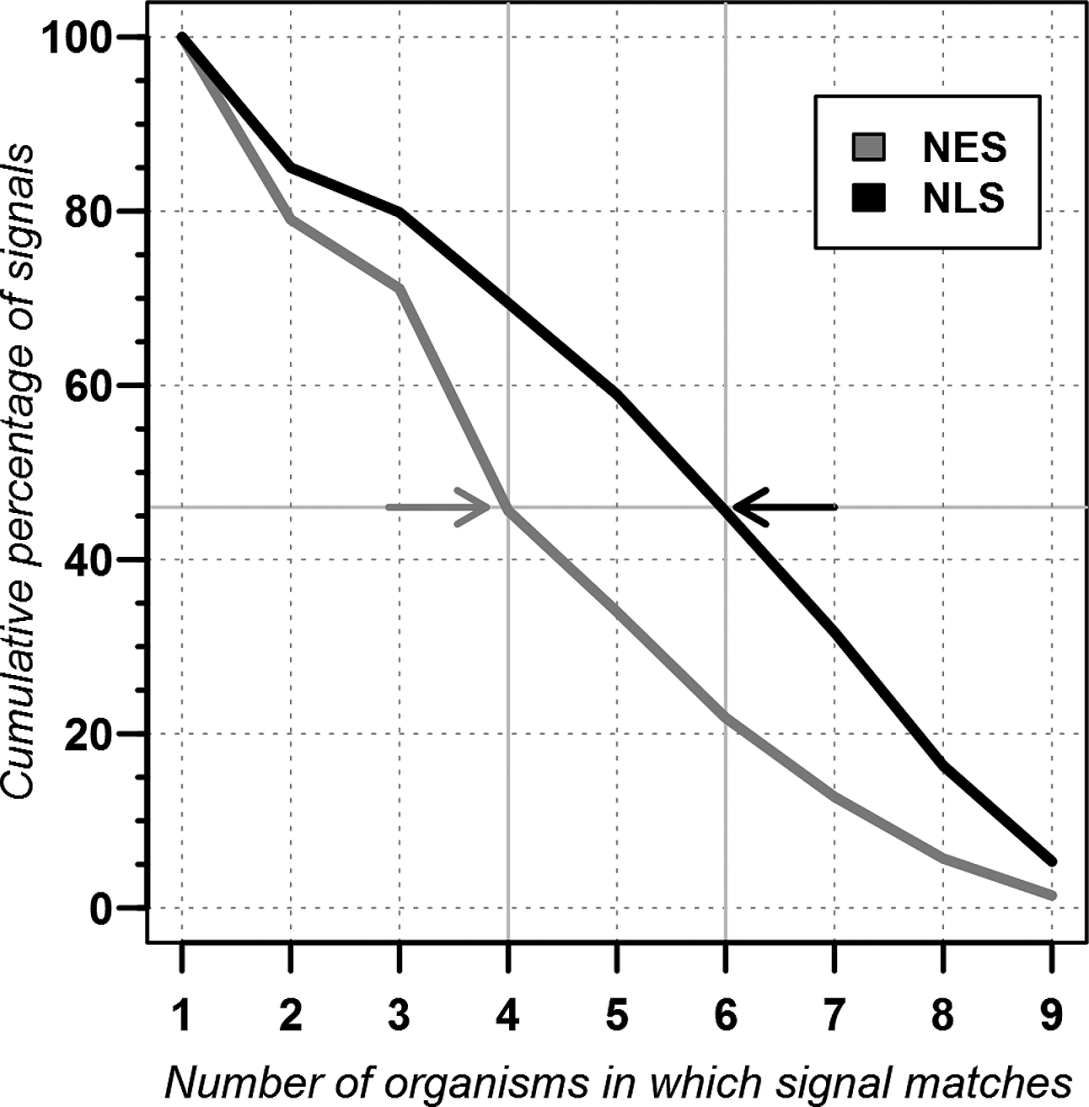
NES and NLS common to multiple organisms. The graph shows the cumulative percentage of NES and NLS found in at least one protein from the nine organisms used in Tables 1–2 (*Arabidopsis thaliana, Caenorhabditis elegans, Drosophila melanogaster, Homo sapiens, Mus musculus, Oryza sativa, Rattus norvegicus, Schizosaccharomyces pombe* and *Saccharomyces cerevisiae*). Hundred percent corresponds to 353 NES and 2180 NLS contained in NLSdb. For instance, 46% of the NES and 46% of the NLS matched in at least four and six organisms, respectively (arrows).

Our *in silico* mutagenesis protocol succeeded in rendering signals that are found in many organisms. For instance, 46% of the NES matched in at least four of nine model organisms, and 46% of the NLS matched in six or more (Figure 2 arrows and Table 1). Only about 1% of all NES and 5% of all NLS matched in all nine organisms.

### NLSdb web interface

In addition to updating the dataset of NES and NLS, we also re-designed the NLSdb web interface. It now supports a wider range of different query types as well as batch queries, i.e. submitting more than one query at a time. Queries can be directly inputted as text or uploaded as a file.

#### Searching proteins for NES and NLS

Users can submit protein sequences to find potential NES and NLS. The server accepts sequences in FASTA format or through their UniProtKB accession numbers. If any NES or NLS match the sequences, NLSdb reports the position and sequence of the hit as well as two confidence scores. These confidence scores are the number of nuclear proteins and families matched by the hit.

#### Searching and evaluating NES and NLS

Users can also submit their own putative NES or NLS signals and either compare those against our dataset or evaluate them on the set of nuclear and non-nuclear proteins. NLSdb returns all NES and NLS that are matched by the query. Submitting a signal for evaluation returns all nuclear and non-nuclear proteins the query is matching.

#### Browsing and downloading the database

NLSdb provides a browse function where users can simply look at all the NES and NLS sequences within NLSdb, as well as those collected from other databases. Each entry includes the following information: (i) type of the signal (possible values: NES or NLS); (ii) number of nuclear proteins and families matched (integers 0-N); (iii) type of evidence for annotation (possible values: experimental, determined by an expert or potential); (iv) database from which motif was extracted (possible values: UniProtKB AC, NESbase, ValidNESs, SeqNLS or *in silico* mutagenesis).

Users can also download all available data: the sets of collected and generated NES and NLS, and the sets of nuclear and non-nuclear proteins. This can be useful if a user wants to check several thousands of sequences on his local machine and does not need all the information provided by NLSdb, e.g. wants only to check if a NES or NLS is present. Currently all downloads are available as comma separated value files.

## CONCLUSION

The substantially updated version of NLSdb makes available NLS and NES that appeared to match about 34 and 6%, respectively, of all the nuclear proteins in nine model organisms (*A. thaliana, C. elegans, D. melanogaster, H. sapiens, M. musculus, O. sativa, R. norvegicus, S. pombe* and *S. cerevisiae*). Before the update, the specific motifs contained in the previous version of NLSdb matched 17-times fewer proteins than the new version. When also allowing error-prone motifs (matching non-nuclear proteins), the old version covered 1.6-times fewer proteins than the new version. The new NLSdb contains about 7-times as many potential signals that help to understand the molecular mechanism of nuclear transport for many uncharacterized proteins. Along with a complete redesign of the interface, this overhauled resource might help in the design and follow up analysis of many experiments.

## DATA AVAILABILITY

The complete sets of nuclear and non-nuclear proteins, as well as the sets of collected and potential NES and NLS, is available at the NLSdb website: https://rostlab.org/services/nlsdb/.
